# Phytochemical Pharmacokinetics and Bioactivity of Oat and Barley Flour: A Randomized Crossover Trial

**DOI:** 10.3390/nu8120813

**Published:** 2016-12-15

**Authors:** Caleigh M. Sawicki, Diane L. McKay, Nicola M. McKeown, Gerard Dallal, C. -Y. Oliver Chen, Jeffrey B. Blumberg

**Affiliations:** 1 Nutritional Epidemiology, Jean Mayer USDA Human Nutrition Research Center on Aging at Tufts University, Boston 02111, MA, USA; caleigh.sawicki@tufts.edu (C.M.S.); nicola.mckeown@tufts.edu (N.M.M.); 2 Antioxidants Research Laboratory, Jean Mayer USDA Human Nutrition Research Center on Aging at Tufts University, Boston 02111, MA, USA; diane.mckay@tufts.edu (D.L.M.); oliver.chen@tufts.edu (C.-Y.O.C.); 3 Biostatistics Unit, Jean Mayer USDA Human Nutrition Research Center on Aging at Tufts University, Boston 02111, MA, USA; jerry.dallal@tufts.edu

**Keywords:** oat, barley, bioactives, whole grain, polyphenols, phenolic acids, tocols, antioxidant

## Abstract

While dietary fiber plays an important role in the health benefits associated with whole grain consumption, other ingredients concentrated in the outer bran layer, including alkylresorcinols, lignans, phenolic acids, phytosterols, and tocols, may also contribute to these outcomes. To determine the acute bioavailability and pharmacokinetics of the major phytochemicals found in barley and oats, we conducted a randomized, three-way crossover trial in 13 healthy subjects, aged 40–70 years with a body mass index (BMI) of 27–35.9 kg/m^2^. After a two-day run-in period following a diet low in phytochemicals, subjects were randomized to receive muffins made with either 48 g whole oat flour, whole barley flour, or refined wheat flour plus cellulose (control), with a one-week washout period between each intervention. At the same time, an oral glucose tolerance test was administered. In addition to plasma phytochemical concentrations, glucose and insulin responses, biomarkers of antioxidant activity, lipid peroxidation, inflammation, and vascular remodeling were determined over a 24-h period. There was no significant effect on acute bioavailability or pharmacokinetics of major phytochemicals. Administered concurrently with a glucose bolus, the source of whole grains did not attenuate the post-prandial response of markers of glucoregulation and insulin sensitivity, inflammation, nor vascular remodeling compared to the refined grain control. No significant differences were observed in the bioavailability or postprandial effects between whole-oat and whole-barley compared to a refined wheat control when administered with a glucose challenge. These null results may be due, in part, to the inclusion criteria for the subjects, dose of the whole grains, and concurrent acute administration of the whole grains with the glucose bolus.

## 1. Introduction

Higher consumption of whole-grain foods has been linked to a lower risk of type 2 diabetes mellitus (T2DM), cardiovascular disease (CVD) risk, and mortality in large prospective cohorts [1,2,3,4,5,6]. While dietary fiber is a predominant nutritional attribute of whole grains, there are a host of potential phytochemicals present in whole-grains that might confer metabolic health benefits [7,8]. These phytochemicals are located principally in the outer bran layer and include alkylresorcinols, flavonoids, lignans, phenolic acids, phytosterols, and tocols (tocopherols and tocotrienols). For many food products, whole grains undergo varying degrees of processing that may lead to an improvement in the bioavailability of its constituent phytochemicals [9,10]. Once absorbed, these phytochemicals are metabolized and may contribute through both direct and synergistic pathways to impact health via anti-inflammatory, antioxidant, and/or anti-proliferation effects [11].

β-glucan is the predominant soluble fiber found in oats and barley and has been shown to reduce serum cholesterol and improve post-prandial insulin and glucose responses in healthy and diabetic adults [12,13]. However, it has been suggested these and other health benefits of whole grains may be the result of a synergistic effect of the fiber and the constituent phytochemicals [14]. The major bioactives in barley include phenolics, tocols and folate, while those in oats include tocopherols and tocotrienols, phenolic acids, sterols, selenium and avenanthramides. To date, no controlled metabolic studies have determined the acute bioavailability and pharmacokinetics of oats and barley flour or considered the post-prandial effect of these phytochemicals on markers of metabolic, inflammatory and oxidative stress, in response to an oral glucose tolerance test (OGTT).

The primary aim of this study was to determine the acute (24-h) bioavailability and pharmacokinetics of the major phytochemicals found in whole barley and oats. The secondary aim was to determine the effect of these bioactives on selected biomarkers associated with risk of T2DM and/or CVD. We designed a study whereby a test meal was administered concurrently with an oral glucose tolerance test (OGTT) to induce acute metabolic dysregulation and an acute increase in several biomarkers of cardiometabolic disease in overweight or mildly obese, middle-aged adults. In comparison to refined wheat flour, we hypothesized that consumption of either whole oat flour or whole barley flour, in conjunction with an OGTT, would attenuate the postprandial response of: (1) glucoregulation and insulin sensitivity; (2) inflammation; (3) vascular remodeling; and (4) antioxidant activity and oxidative stress.

## 2. Materials and Methods

### 2.1. Subject Eligibility

Fourteen overweight or mildly obese, metabolically at-risk, nonsmoking men and postmenopausal women, aged 40–70 years with a body mass index (BMI) of 27.0–35.9 kg/m^2^, were recruited from the Boston area. Postmenopausal status in women was defined as the absence of menses for ≥1 year. The exclusion criteria used to screen for eligibility included: (a) presence of cardiovascular, endocrine, gastrointestinal, and renal diseases; (b) use of estrogen, with or without progesterone; (c) use of medications known to affect lipid metabolism; (d) use of medications known or suspected to influence blood pressure (BP); (e) gastrointestinal diseases and conditions or medications influencing gastrointestinal absorption; (f) chronic kidney disease; (g) endocrine disorders including diabetes and untreated thyroid disease; (h) rheumatologic disorders; (i) active treatment for cancer of any type (except basal cell carcinoma) ≥1 year; (j) regular use of oral steroids; (k) systolic blood pressure >150 mmHg and/or diastolic blood pressure >95 mmHg; (l) regular use of any dietary supplements within ≤30 day; (m) usual daily ethanol intake of ≥2 drinks; cigarette smoking and/or nicotine replacement use; (n) and laboratory blood or urine biochemistries outside of normal ranges. The study design was approved by the Institutional Review Board of Tufts University Health Sciences Campus and Tufts Medical Center. All participants signed a written informed consent agreement before participating. This study was registered with the public registry ClinicalTrials.gov (ID # NCT01303562). (Trial Registration: ClinicalTrials.gov NCT01303562)

### 2.2. Study Design and Intervention

A randomized, double-blind, placebo-controlled three-way crossover trial was conducted to evaluate the postprandial effects of the phytochemicals present in oats and barley on selected measures of antioxidation (α-tocopherol, γ-tocoperhol, total antioxidant capacity) and phenolic acids (including benzoic, caffeic, *p*-coumaric, ferulic, phenylacetic, protocatechuic, sinapic, and vanillic acids); markers of inflammation including high sensitivity C-reactive protein (hsCRP), interleukin (IL)-6 IL-8 and tumor necrosis factor-alpha (TNF-α); markers of glucoregulation including fasting glucose, insulin, leptin and adiponectin; and a marker of vascular remodeling, matrix metalloprotein 9 (MMP-9). These determinations were made following consumption of a muffin containing barley or oat whole grains or refined wheat and a concurrent OGTT. Subjects were asked to refrain from eating foods containing high amounts of alkylresorcinols, phenolic acids, phytosterols, tocols, and other polyphenols for 2 days prior to each study visit. These foods included any whole grains, legumes, beans, certain fruits, berries, vegetables, juices, nuts, seeds, vegetable oils, herbs, spices, tea, herbal teas, coffee, cocoa, chocolate, and wine. To increase compliance with these directions, subjects were provided with low polyphenol meals, which were consumed on the day prior to each study visit. The purpose of the dietary restrictions was to reduce any residual dietary phenolic compounds in the body, which are typically cleared from blood and urine within 48 h of consumption.

The trial consisted of three acute (24-h) interventions in which subjects were randomized to receive a one-time dose of 48 g of whole grain in two small muffins (24 g flour/muffin). The flour was either whole oat flour, whole barley flour, or refined wheat flour (placebo). There was a one-week washout period between interventions. The dose was based on the amount of grain typically included in a breakfast meal consisting of a bowl of ready-to-eat cereal. During each intervention, an OGTT was performed on each subject for 3 h following the consumption of a glucose bolus (75 g in 300 mL water) concomitantly with the test muffins. The oat and barley flours were provided by the Kellogg Company (Battle Creek, MI, USA), and the refined wheat flour was purchased at a local grocery. Each muffin on average provided 48.7 g flour, ~310 kcal, 8.2 g total fat, 7.8 g protein, and 52.4 g carbohydrate (Table 1). Although the oat and barley muffins provided the same amount of total fiber (4.9 g each), the proportion of soluble fiber was higher in the barley compared to the oat muffin (3.2 vs. 2.4 g, respectively). The placebo muffin had less total fiber (3.2 g) but a higher proportion of insoluble fiber, due to the added cellulose (2.4 g). Cellulose was added to the placebo muffin to adjust the total fiber content so it more closely resembled that of the whole grain muffins; however, because of its properties (insoluble, poorly fermented), cellulose has no effect on blood lipids or glucose and is, therefore, often used as a placebo in clinical trials [15].

Randomization was stratified by gender according to a computer-generated list. Study personnel were blinded to the treatment assignment for the duration of the intervention and sample analysis. The only exception was the study dietitian who was responsible for distributing the test breakfasts, dietary instructions, and meals to eligible subjects at randomization, and assessing compliance. During each visit, subjects reported to the Clinical and Translational Research Center (CTRC) at Tufts Medical Center after fasting for 12 h. At each visit, subjects were queried regarding interval changes in health, as well as use of prescription medications, tobacco, and dietary supplements.

Prior to administering the test breakfast (muffins plus OGTT), fasting blood and urine (24 h) samples were collected for baseline measurements. The test breakfast was administered under close observation by the study staff. No other food or beverage was provided at this time. Following administration of the test breakfast, blood samples were collected via indwelling venous catheter at 0.5, 1, 1.5, 2, 3, 4, 6, 8, 10, and 24 h. Lunch and dinner meals prepared by the research center kitchen were low in phenols and polyphenols and were provided 5 h and 10 h post-administration. At the end of 10 h, subjects were allowed to leave the CTRC and returned the following morning after having fasted for 12 h. Fasting blood was collected within 24 h of administering the test breakfast. Vital signs, including blood pressure, temperature, pulse, and respiration rate, were monitored regularly for the 10 h following consumption of the test breakfast.

### 2.3. Sample Size

In a placebo-controlled, three-way crossover trial with six healthy older adults, Chen et al. [16] observed significantly increased plasma levels of the oat polyphenols (avenanthramides A, B, and C) within 2 h of consumption. Since our goal of determining the pharmacokinetic parameters related to the consumption of flaked/rolled oats and barley is compatible with this previous study, a sample size of at least six subjects is justified. However, the oat product used by Chen et al. [16] differs from that used in this proposed study as it was enriched in avenanthramides via an industrial concentration procedure. Thus, we determined a sample size of 12 subjects to be reasonable for our intervention. As mentioned above, *n* = 14 subjects were initially recruited to account for potential dropouts.

### 2.4. Sample Collection and Preparation

Collected samples were assessed for the ferric reducing ability of plasma (FRAP), resistance of low density lipoprotein cholesterol (LDL) to Cu^2+^-induced oxidation (LDL oxidation), total thiols, glucose, insulin, leptin, adiponectin, hsCRP, IL-6, IL-8, TNFα, and MMP-9. Blood samples for the analysis of FRAP and LDL oxidation were collected in EDTA-containing evacuated tubes and centrifuged within 15 min of drawing (1000 × *g*, 15 min, 4 °C) with a SUR-Sep cap (Organon Teknika, Durham, NC, USA). Blood samples for the remaining analytes were collected in serum separator tubes and processed similarly. Plasma samples for the analysis of LDL oxidation were prepared by adding 111 µL of 6% sucrose solution to 1 mL plasma, and stored at −80 °C for no longer than eight weeks before analysis. All samples were stored at −80 °C until analysis. All samples for each participant were analyzed within the same run for every assay performed.

### 2.5. Biochemical Analyses

#### 2.5.1. Tocopherols

Plasma α- and γ-tocopherols were quantified using an ultra-high pressure liquid chromatography (UHPLC) fluorescence detection method according to Liu et al. [17]. Briefly, tocopherols in 200 µL plasma were added with α-tocopheryl acetate as the internal standard and then extracted twice with hexane. The hexane fractions were combined, dried under N_2_ gas, and reconstituted with ethanol for tocopherol analysis using an Nexera UHPLC System (Shimadzu, Columbia, MD, USA), equipped with a LC-30AD pump, a SIL-30AC autosampler, a CTO-30A column oven, a SPD-M20A photodiode array (PDA) detector (monitoring at 284 nm), a Shimadzu RF-10A XL fluorescence detector (monitoring at Ex 297 nm and Em 328 nm), and a Kinetex^®^ 2.6 μm C18 100 Å, LC Column 50 × 3 mm (Phenomenex Inc., Torrance, CA, USA). Tocopherols in plasma samples were calculated using standard curves constructed with authentic standards and adjusted to the internal standard. The intra-day coefficients of variance (CV) for α- and γ-tocopherols was 7.1 and 7.6%, respectively, and the inter-day CV was 13% and 12%.

#### 2.5.2. Alkylresorcinols

Plasma alkylresorcinols were determined according to our previously published method [18]. Briefly, individual alkylresorcinol homologues C19:0, C21:0, and C23:0 in plasma were extracted using diethyl ether after protein precipitation using ethanol. Alkylresorcinol C20:0 was administered as the internal standard. Alkylresorcinols in the resulting extract were further purified using a Waters Oasis Max cartridge, followed by derivatization using trifluoroacetic acid and quantification using gas chromatography–mass spectrometry (GC-MS) in negative chemical ionization, selected ionization monitoring modes and an Agilent HP-5 capillary column (30 m × 0.25 mm × 0.25 μm) (Agilent, Santa Clara, CA, USA). Plasma concentrations of alkylresorcinols were calculated using standard curves constructed with authentic standards spiked into quality control plasma with adjustment to the internal standard. Intraday CV for C19:0, C21:0, and C23:0 were 0.8, 2.0 and 3.3%, respectively; inter-day CV were 5.3%, 8.0%, and 12.4%, respectively.

#### 2.5.3. Phenolic Acids

Plasma phenolic acids were determined according to Chen et al. [19] for HPLC analysis using an ESA CoulArray System (ESA, Inc., Chelmsford, MA, USA). Analyte separation was achieved using a Zorbax ODS C18 column (4.6 × 250 mm, 3.5 μm). Quantification of phenolic acids and flavonoids in unknown samples were calculated based on standard curves constructed using authentic standards and adjustment to the internal standard (4′-hydroxy-3′-methoxyacetophenone).

#### 2.5.4. Antioxidant Capacity

The ex vivo resistance of LDL to Cu^2+^-induced oxidation was determined by monitoring the formation of conjugated dienes at 37 °C over 3 h with a Shimadzu UV1601 spectrophotometer at an absorbance of 234 nm according to Chen et al. [19]. The results are expressed as lag time (min). The FRAP value of whole plasma was determined by the spectrophotometric method of Benzie and Strain [20]. Total thiols (-SH moieties) in plasma were determined according to the spectrometric method of Hu [21].

#### 2.5.5. Inflammation and Vascular Remodeling

High sensitivity CRP (hsCRP) in serum was determined using the Randox Metabolic Syndrome Array II using a biochip array multiplex technology (Kearneysville, WV). IL-6, IL-8, and TNF-α were measured using the Randox Cytokine Array I and MMP-9 using the Randox Cytokine Array IV. All assays were performed according the manufacturer’s instructions and analyzed using Evidence Investigator (Randox, Kearneysville, WV, USA).

#### 2.5.6. Glucoregulation and Insulin Sensitivity

Serum glucose was measured by an enzymatic couple method using an AU400 clinical chemistry analyzer (Beckman Coulter, Inc., Brea, CA, USA) as specified in the manufacturer’s procedural documentation, with intra- and inter-assay CV of 2.0% and 3.2%, respectively. Serum insulin was measured using a solid phase sandwich enzyme linked-immuno-sorbent assay kit procedure, (Invitrogen Human Insulin ELISA kit, Camarillo, CA, USA) as specified in the manufacturer’s procedural documentation, with intra- and inter-assay CV of 5.4% and 8.5%, respectively. Leptin and adiponectin in serum was determined using a Randox Metabolic Syndrome Array I and II, respectively, according to the manufacturer’s instructions.

### 2.6. Statistical Analyses

All results are presented as mean ± standard error. For each of the selected biomarkers, maximum plasma concentration (C_max_), time to reach maximum plasma concentration (T_max_), and area under the time-course curve expressed as a percent of baseline (AUC%) were calculated. The AUC% was calculated for each biomarker using the linear trapezoidal integration method [22] with the percentage of change in concentration at each time point (0–24 h). Repeated measures analysis of variance (ANOVA) determined the effects of treatment, adjusted for multiple comparisons with the Tukey-Kramer method. All ANOVAs were additionally adjusted for visit to control for within subject variability. Wilcoxon signed rank sum test was used to determine significant differences between C_max_ and baseline concentrations, and single sample t-test was used to determine if AUC% was significantly different from zero. Tests were performed with and without outliers, but no difference in results was observed. *P* values ≤ 0.05 were considered statistically significant. Statistical analyses were performed using the SAS statistical software package, version 9.3 (SAS Institute Inc., Cary, NC, USA).

## 3. Results

Fourteen subjects were recruited and followed from September 2010 to April 2011. One subject dropped out, due to lack of interest, following the first visit. Thirteen subjects (8 males/5 females), mean age 53.1 ± 7.0 year, BMI 32.3 ± 3.1 kg/m^2^, with fasting total cholesterol and triglyceride levels of 208.2 ± 28.1 and 110.9 ± 53.8 mg/dL, respectively, completed the trial. Of the 13 subjects, 9 (69.2%) had elevated total cholesterol (≥200 mg/dL), and 4 (30.8%) had elevated triglycerides (≥150 mg/dL). Table 2 presents characteristics of the 13 subjects. No untoward effects were reported.

Table 3 shows the results of the pharmacokinetics of the tocopherols, phenolic acids, and alkylresorcinols. Overall, these bioactives tended to increase from baseline (except for sinapinic acid), but there were no statistically significant differences between treatment groups. C_max_ for 4-OH-3-MeOH-phenylacetic acid, caffeic acid, and sinapinic acid was not significantly different from baseline in any of the muffins. No significant difference was observed in the C_max_ or T_max_ for either barley or oat muffins compared to the white flour muffin.

Table 4 presents the observed effects on selected biomarkers associated with risk of T2DM and CVD. No statistically significant differences between groups were observed over 0–24 h with biomarkers of antioxidant capacity, inflammation, vascular remodeling or glucoregulation. In addition, no statistically significant results were noted for any of the above measures when examining 0–4 h or 0–10 h periods (data not shown). hsCRP was unchanged between baseline and C_max_ after administration of all three muffins.

## 4. Discussion

The phytochemical ingredients of whole grains may contribute to their associated health benefits [11], but metabolic studies examining their bioavailability and bioactivity in humans are limited. The present study examined whether an acute intake of muffins made with whole oat or barley flour would increase plasma phytochemical status and improve some biomarkers of CVD risk. A single dose of whole oat or barley flour had no effect on the plasma concentration of alkylresorcinols, phenolic acids or tocopherols in overweight and obese adults. Markers of antioxidant capacity were also unchanged. When administered concurrently with a glucose bolus, neither source of whole grains attenuated the postprandial response of markers of glucoregulation, inflammation or vascular remodeling when compared to the control intervention of refined wheat grain.

The phytochemical content varies considerably within and between major cereals [23], and although some grains may contain high amounts of these compounds, many, including phenolic acids and flavonoids, are poorly bioaccessible because of their tight conjugation to the cell wall matrix [24]. The outer structure of the grains, including the pericarp seed coat and aleurone layer, generally contain much higher phytochemical concentrations than the germ and endosperm compartments, and the ultimate bioavailability of these phytochemicals may depend greatly on the degree and manner in which the grain was processed before consumption [10]. Few studies have examined the bioavailability of phenolic acids and polyphenols from oats and barley in humans. Previously, we reported that oat avenanthramides are bioavailable in humans and hamsters when much larger doses than tested here were administered [16,19]. To date, no clinical trial has examined the bioavailability of phenolic acids in barley or oats, in which ferulic acid content ranges from 10–50 mg/100 g (~2.4 mg/dose in the present study) [25]. Ferulic acid is a predominate phenolic acid in whole wheat, and its bioavailability has been reported in a few trials with products made with whole wheat plus wheat bran or only whole wheat or wheat bran [9,14,26]. Vitaglione et al. [14] found serum ferulic acid increased 80% after eight weeks of daily consumption of 70 g whole wheat products but was unchanged when refined wheat products were consumed. In an acute study, consumption of ~93 g wheat bran led to ≤2% increase in plasma total phenols 60 min post-ingestion [27]. Although the phenolic content of the muffins provided to the study participants were not measured in the present study, the concentration was likely much lower than administered in other studies; thus, we speculate that unchanged status of circulating phenolic acids may be attributed mostly to the low dose employed here.

The evidence relating long-term whole-grain interventions with cardiometabolic risk factors is mixed, with some studies showing a beneficial effect [14,28,29,30] while others present a null outcome [31,32]. Only a few studies have tested whether the cardiometabolic effects of whole grains may be partly attributed to their phytochemical profile. In the present study, we found no difference in the LDL resistance against Cu^2+^-induced oxidation after ingestion of either the oat or barley muffin. Other intervention studies [31,33,34] have compared the effect of whole grain rye or wheat to refined grains on plasma and urinary biomarkers of lipid peroxidation and also observed no improvement over a longer period of consumption (2–6 weeks) in healthy, overweight adults [31] or normal weight adults [33,34]. Similarly, neither the oat nor barley muffin changed plasma levels of FRAP and total thiol or other measures of total antioxidant capacity. Previously, we found that an oat extract containing 1 g avenanthramide-enriched mixture increased circulating reduced glutathione; however, this dose greatly exceeds the avenanthramide content of naturally occurring in whole grain oats [16]. Consistent with our results, Seidel et al. [35] found no change in plasma FRAP in male smokers and non-smokers fed a high α-tocopherol bread (rich in inulin, linseed, and soya fiber) or a fiber-rich wheat-rye control bread for five weeks. Indeed, the majority of intervention studies comparing whole versus refined grains on pro-inflammatory markers, including CRP, IL-6, IL-8 and TNF-α, have observed no statistically significant benefit [30,31,36,37,38].

Despite the expected difference in the concentration of phytonutrients (α- or γ-tocopherol, benzoic, caffeic, *p*-coumaric, ferulic, phenylacetic, protocatechuic, sinapic, and vanillic acids) between whole and refined grain muffins, the relatively small concentrations found in either muffin may be insufficient to elicit any cardiometabolic effect. In summary, our results in this acute intervention suggest that the effect of phenolic compounds and/or their interactions with dietary fiber may be insufficient in the context of an OGTT to increase phytochemical status and attenuate the glucose-induced metabolic stress. Our results are consistent with the conclusion of Belobrajdic and Bird [24] that there may be only a minor or negligible effect on biomarkers of oxidative stress or antioxidant activity in response to whole grain cereal consumption.

In acute feeding studies that have examined the effect of whole grain oats and barley on post-prandial insulin and glucose responses [39,40,41], the observed physicochemical effect has been attributed to β-glucan, a soluble, viscous/gel-forming dietary fiber [42]. The soluble fiber content (i.e., β-glucan) of the barley and oat muffins in the present intervention ranged from 2.4–3.2 g. Interestingly, Kim et al. [43] reported a barley breakfast providing ≥10 g β-glucan was necessary to observe a beneficial effect on glycemic response in obese women who were at increased risk of developing insulin resistance. In addition to the effect of dose, the effect of cooking or baking of whole grains may alter the physiological effects of soluble fiber; e.g., Kerckhoffs et al. [44] found when oats are baked into bread or cookies, the β-glucan naturally found in oats becomes less viscous than raw β-glucan. This reduced viscosity was linked to a reduction in the ability of β-glucan present in oats and barley to modify glucose and cholesterol absorption in the gastrointestinal tract. Similarly, cooking can reduce the concentrations of phytochemicals present in whole grain foods [45]. In the present study, the processing of whole grains into flour and baking into muffins could have altered the structure and content of the grains such that the fiber and phytochemicals were less effective at attenuating cardiometabolic risk factors than expected.

A strength of this study was its design which kept the macronutrient composition of the test muffins comparable and the addition of dietary fiber to the refined wheat muffin control so that any observed changes would have been most likely due to differences in their phytochemical and/or micronutrient profiles rather than the dietary fiber. Nonetheless, there are potential limitations of our study design that may partly explain the lack of an observed effect of whole grain oats or barley on the acute bioavailability of their constituent phytochemicals. Firstly, the study population was a convenience sample of metabolically at-risk individuals, and, as such, generalizability would likely be limited to metabolically at risk populations with a greater predisposition to systemic inflammation, oxidative stress, and insulin resistance than younger, leaner adults. The relatively small sample size may have also contributed to the observed non-significant results. Secondly, our study design included an OGTT administered concurrently with the test muffins, an approach that was intended to stimulate an acute metabolic dysregulation in order to measure an attenuation of the physiological response to this stress. However, this glucose bolus may have overwhelmed any subtle effect of phytochemicals in the whole grains on the postprandial glycemic response, especially given that the digestion and subsequent metabolic and physiologic responses to free glucose differs from that in a solid food matrix. Further, there is high intra- and inter-personal variability in both postprandial glucose responses, especially in ‘at-risk individuals’, and phytochemical metabolism [46,47,48]. The processing and cooking of the whole grains may have influenced acute bioefficacy of their constituent phytochemicals and fiber, and the contents of phytochemicals in the muffins were not measured prior to consumption and may be too low to elevate their circulating concentrations to the detectable levels. Lastly, measurements during 11–23 h and beyond 24 h were not collected and, therefore, significant observations during these time frames may have been missed. Nonetheless, we feel this study fills a gap in research on whole grains, as few other acute feeding studies have considered the postprandial responses of phytochemicals on biomarkers of inflammation and oxidative stress.

## 5. Conclusions

In conclusion, we found no statistically significant differences in the bioavailability or postprandial metabolic effects of whole oat and whole barley compared to a refined wheat control when administered in muffins together with a glucose bolus challenge. Future studies should consider using whole grain varieties with minimal processing that deliver the highest possible intake of bioaccessible phytochemicals in the context of whole or functional foods.

## Figures and Tables

**Table 1:** Macronutrient composition per two test muffins containing ~48 g whole grain flour.

Nutrient	Wheat	Barley	Oat
Energy (kcal)	312	302	316
Total fat (g)	7.2	7.5	9.9
Total protein (g)	7.3	7.4	8.6
Total carbohydrate (g)	55.9	52.4	48.9
Total fiber (g)	3.2	4.9	4.9
Soluble (g)	0.8	3.2	2.4
Insoluble (g)	2.4	1.7	2.5

**Table 2:** Subject Characteristics (*n* = 13).

Gender	Age (Year)	Cholesterol (mg/dL)	TG (mg/dL)	SBP (mmHg)	DBP (mmHg)	Height (cm)	Weight (kg)	HR (bpm)
F	49	209	82	125	77	162.8	89.9	57
M	62	215	74	125	79	183.5	97.5	55
F	65	249	52	146	80	154.5	69.5	62
F	58	245	202	142	85	157.8	81.3	85
F	53	211	77	108	74	157	73.6	86
F	53	160	72	113	65	157.5	76.3	63
M	44	172	79	156	74	176	93	105
M	52	209	127	145	95	176.4	109.6	95
M	52	218	63	143	89	174.2	90	80
M	44	211	192	117	64	172.3	88.5	63
M	60	244	84	126	79	177	95.4	74
M	55	181	185	115	65	180.4	91.8	61
M	43	183	153	129	87	178.8	92.4	84

Abbreviations: TG, triglycerides; SBP, systolic blood pressure; DBP, diastolic blood pressure; HR, heart rate; bpm, beats per minute.

**Table 3:** Plasma pharmacokinetics of phytochemicals in humans consuming muffins made with whole oat, whole barley or refined wheat flour (0–24 h).

Phytonutrients	Baseline Mean (SE)	C_max_ Mean (SE)	T_max_ (h) Mean (SE)	AUC (%) Mean (SE)
Tocopherols				
α-Tocopherol (µg/mL)				
White flour	12.94 (1.01)	13.69 (1.00) *	13.87 (3.18)	2350.91 (79.53)
Barley	12.99 (0.96)	14.44 (0.99) *	14.93 (3.12)	2430.42 (77.56)
Oats	12.12 (0.67)	14.03 (0.98) *	16.00 (3.04)	2600.90 (74.45)
γ-Tocopherol (µg/mL)				
White flour	1.95 (0.18)	2.12 (0.26) *	5.03 (3.38)	2152.92 (104.95)
Barley	1.84 (0.21)	2.15 (0.26) *	8.71 (3.33)	2222.35 (102.82)
Oats	1.92 (0.22)	2.24 (0.26) *	12.11 (3.25)	2332.25 (99.64)
Alkylresorcinols				
C19 (ng/mL)				
White flour	8.66 (6.21)	16.02 (4.40) *	10.09 (1.50)	4256.37 (782.41)
Barley	4.68 (1.83)	10.53 (4.20) *	7.69 (1.42)	3740.56 (737.42)
Oats	3.49 (1.19)	8.66 (3.99) *	8.14 (1.33)	5294.69 (688.65)
C21 (ng/mL)				
White flour	15.67 (8.18)	36.35 (8.77) *	6.76 (1.28)	3925.56 (1129.08)
Barley	9.63 (2.15)	26.72 (8.33) *	7.85 (1.21)	4037.47 (1063.76)
Oats	10.73 (2.64)	24.64 (7.87) *	8.68 (1.13)	5100.95 (992.86)
C23 (ng/mL)				
White flour	2.55 (0.90)	10.38 (2.63) *	5.83 (1.50)	7157.18 (1903.26)
Barley	2.68 (0.70)	11.87 (2.55) *	8.06 (1.43)	6630.86(1827.33)
Oats	4.02 (1.93)	8.52 (2.48) *	7.52 (1.34)	7764.84 (1752.87)
Phenolic acids				
3-OH-Benzoic acid (ng/mL)				
White flour	13.57 (3.02)	28.87 (5.69) *	2.51 (1.58)	4323.28 (1261.36)
Barley	21.38 (4.37)	31.73 (5.69) *	4.22 (1.58)	4035.64 (1261.36)
Oats	22.10 (5.87)	26.64 (5.84) *	4.75 (1.73)	2682.94 (1369.57)
4-OH-Benzoic acid (ng/mL)				
White flour	372.06 (68.21)	434.52 (75.78) *	2.92 (2.37)	1829.61 (169.20)
Barley	305.53 (53.85)	377.15 (75.78) *	8.57 (2.37)	2226.02 (169.20)
Oats	416.54 (85.66)	501.80 (78.21)	4.83 (2.57)	1695.74 (183.33)
Caffeic acid (ng/mL)				
White flour	6.41 (0.16)	7.20 (0.51)	2.23 (1.53)	2412.54 (36.01)
Barley	6.25 (0.00)	6.64 (0.51)	0.49 (1.53)	2448.38 (36.01)
Oats	6.33 (0.09)	6.55 (0.53)	3.00 (1.68)	2393.87 (39.00)
p-Coumaric acid (ng/mL)				
White flour	7.83 (0.55)	17.23 (4.45) *	3.71 (2.26)	3768.92 (795.69)
Barley	9.76 (2.73)	14.57 (4.45) *	6.23 (2.26)	2829.41 (795.69)
Oats	12.78 (3.82)	15.47 (4.54) *	5.00 (2.39)	2356.21 (878.18)
Ferulic Acid (ng/mL)				
White flour	27.20 (5.08)	33.44 (8.84) *	2.44 (1.49)	2016.09 (381.85)
Barley	25.25 (3.99)	36.19 (8.84) *	4.85 (1.49)	2725.58 (381.85)
Oats	40.67 (12.11)	50.96 (9.53) *	5.88 (1.64)	2192.43 (421.66)
4-OH-phenylacetic acid (ng/mL)				
White flour	436.74 (44.87)	504.86 (42.21) *	7.93 (2.53)	2226.48 (156.92)
Barley	401.60 (38.91)	476.70 (42.21) *	6.87 (2.53)	2243.40 (156.92)
Oats	381.08 (40.11)	469.17 (44.86) *	5.79 (2.80)	2174.89 (167.74)
4-OH-3-MeOH-phenylacetic acid (ng/mL)				
White flour	21.98 (11.92)	23.58 (8.23)	0.80 (0.53)	2406.07 (172.43)
Barley	13.96 (5.06)	15.58 (8.23)	0.14 (0.53)	2241.05 (172.43)
Oats	13.48 (3.61)	17.72 (8.92)	1.08 (0.58)	2186.59 (186.03)
Protocatechuic acid (ng/mL)				
White flour	37.31 (13.38)	44.82 (28.12) *	5.96 (2.52)	2292.83 (527.21)
Barley	34.47 (12.32)	59.16 (28.12) *	8.64 (2.52)	3381.82 (527.21)
Oats	67.52 (38.40)	89.18 (30.14) *	6.98 (2.67)	2577.70 (577.78)
Sinapinic acid (ng/mL)				
White flour	8.27 (1.09)	11.01 (2.43)	0.46 (0.78)	2361.71 (77.00)
Barley	8.00 (0.79)	9.28 (2.43)	1.66 (0.78)	2452.80 (77.00)
Oats	8.22 (0.83)	9.22 (2.61)	1.76 (0.85)	2560.90 (84.05)
Vanillic acid (ng/mL)				
White flour	166.49 (27.06)	219.87 (29.70) *	3.37 (1.56)	2010.91 (198.18)
Barley	148.35 (24.18)	184.75 (29.70) *	5.79 (1.56)	2039.76 (198.18)
Oats	161.87 (27.83)	169.59 (30.63) *	1.96 (1.68)	2161.16 (219.06)

*C_max_: maximum plasma concentration; T_max_: time to reach maximum plasma concentration; AUC (%): area under the time-course curve expressed as a percent of baseline; C_max_ significantly different from baseline, *p* ≤ 0.05, Wilcoxon signed rank.

**Table 4:** Postprandial response of biomarkers associated with cardiovascular disease (CVD) and/or type 2 diabetes mellitus (T2DM) risk in humans consuming muffins made with whole oat, whole barley or refined wheat flour (0–24 h).

	Baseline Mean (SE)	C_max_ Mean (SE)	T_max_ (h) Mean (SE)	AUC (%) Mean (SE)
Total Antioxidant Capacity				
Ferric reducing ability of plasma (FRAP) (µmol/L)				
White flour	408.93 (27.44)	424.91 (29.88) *	9.80 (3.25)	2252.67 (67.30)
Barley	399.73 (23.49)	423.62 (29.26) *	3.81 (3.17)	2227.55 (65.44)
Oats	390.41 (24.49)	428.45 (28.45) *	6.50 (2.92)	2405.67 (61.32)
Total thiols (mmol/L)				
White flour	0.28 (0.01)	0.33 (0.02) *	7.13 (1.94)	2469.79 (90.39)
Barley	0.32 (0.02)	0.34 (0.01) *	1.60 (1.71)	2135.75 (80.49)
Oats	0.29 (0.01)	0.34 (0.02) *	3.52 (2.16)	2466.64 (100.52)
Lag Time of LDL oxidation (min)				
White flour	100.23 (6.26)	125.16 (8.99) *	8.68 (2.07)	2406.08 (86.09)
Barley	102.40 (6.06)	126.32 (9.07) *	3.63 (2.14)	2394.40 (88.91)
Oats	107.52 (8.59)	132.40 (9.06) *	5.22 (2.14)	2392.58 (88.90)
Inflammation				
hsCRP (mg/L)				
White flour	5.90 (0.59)	12.38 (3.74) *	9.62 (3.58)	2858.15 (374.92)
Barley	11.08 (3.28)	13.54 (3.28) *	11.88 (3.17)	2327.15 (340.48)
Oats	7.35 (1.08)	10.38 (3.52) *	9.07 (3.38)	2874.58 (358.53)
IL-6 (pg/mL)				
White flour	3.05 (1.09)	6.03 (1.74) *	13.60 (1.92)	4343.36 (835.79)
Barley	2.84 (0.80)	5.00 (1.64) *	5.77 (1.80)	3255.87 (794.41)
Oats	4.13 (2.36)	7.51 (1.64) *	9.25 (1.80)	4596.24 (794.36)
IL-8 (pg/mL)				
White flour	4.33 (0.05)	5.84 (0.65) *	5.94 (1.99)	2491.11 (305.91)
Barley	4.46 (0.72)	5.70 (0.65) *	6.19 (1.99)	2241.28 (305.91)
Oats	4.15 (0.62)	6.88 (0.63) *	7.87 (1.88)	2941.49 (288.48)
TNF-α (pg/mL)				
White flour	4.33 (0.41)	5.53 (0.72) *	10.84 (2.52)	2683.58 (137.83)
Barley	5.30 (0.58)	5.81 (0.70) *	5.67 (2.38)	2181.28 (129.96)
Oats	5.26 (0.68)	6.30 (0.70) *	8.99 (2.38)	2419.13 (129.96)
Vascular Remodeling				
Matrix Metalloprotein 9 (MMP9) (ng/mL)				
White flour	59.93 (13.91)	151.57 (24.79) *	6.29 (1.28)	4558.91 (563.75)
Barley	69.40 (12.21)	154.13 (24.68) *	5.11 (1.26)	3714.29 (551.07)
Oats	87.0 (17.71)	161.40 (24.68) *	6.32 (1.26)	3178.33 (551.07)
Glucoregulation and Insulin Sensitivity				
Glucose (mg/dL)				
White flour	106.85 (2.14)	187.00 (9.37) *	0.68 (0.10)	383.46 (17.96)
Barley	104.75 (2.83)	186.07 (9.23) *	0.75 (0.10)	391.82 (17.76)
Oats	104.50 (3.35)	196.53 (9.92) *	0.93 (0.11)	396.40 (18.65)
Insulin (IU/mL)				
White flour	22.68 (3.00)	163.24 (17.44) *	1.29 (0.17)	1432.11 (121.44)
Barley	21.07 (2.07)	152.69 (17.44) *	1.23 (0.17)	1429.87 (121.44)
Oats	20.98 (2.53)	157.45 (17.44) *	1.43 (0.17)	1407.78 (121.44)
Leptin (ng/mL)				
White flour	4.12 (1.51)	5.91 (1.60) *	8.78 (1.30)	2637.31 (228.45)
Barley	3.72 (0.91)	5.25 (1.58) *	5.41 (1.12)	2283.19 (205.02)
Oats	3.91 (1.20)	5.81 (1.58) *	7.69 (1.12)	3065.44 (204.98)
Adiponectin (ng/mL)				
White flour	16.83 (3.07)	23.45 (3.77) *	8.92 (2.58)	2842.75 (286.91)
Barley	18.58 (3.56)	20.96 (3.59) *	3.98 (2.30)	2327.23 (273.41)
Oats	18.72 (2.90)	21.32 (3.68) *	7.20 (2.44)	2133.00 (280.96)

*C_max_: maximum plasma concentration; T_max_: time to reach maximum plasma concentration; AUC (%): area under the time-course curve expressed as a percent of baseline; LDL: low density lipoprotein; hsCRP: High sensitivity C-reactive protein; IL: interleukin; TNF-α: tumor necrosis factor-alpha; C_max_ significantly different from baseline, *p* ≤ 0.05, Wilcoxon signed rank.
